# Ketogenic diet and β-hydroxybutyrate in osteoporosis: current progress and controversy

**DOI:** 10.3389/fnut.2025.1508695

**Published:** 2025-01-23

**Authors:** Changfang Luo, Zhuojun Dai, Wanhong He, Yanqiu He, Ping Yang, Mengting Huang, Junle Li, Yong Xu, Wei Huang

**Affiliations:** ^1^Department of Endocrinology and Metabolism, The Affiliated Hospital of Southwest Medical University, Luzhou, Sichuan, China; ^2^Metabolic Vascular Diseases Key Laboratory of Sichuan Province, Luzhou, Sichuan, China; ^3^Sichuan Clinical Research Center for Nephropathy, Luzhou, Sichuan, China; ^4^Sichuan Clinical Research Center for Diabetes and Metabolic Diseases, Luzhou, Sichuan, China

**Keywords:** ketogenic diet, β-hydroxybutyrate, osteoporosis, osteoblast, osteoclast

## Abstract

Diet has been proven to have significant impacts on the pathogenesis and treatment of osteoporosis. This review attempts to elucidate the current progress and controversy surrounding the ketogenic diet (KD) and β-hydroxybutyrate (BHB) in osteoporosis and offers a novel perspective on the prevention and treatment of osteoporosis. The ketogenic diet has been broadly used in the treatment of epilepsy, diabetes, obesity, and certain neoplasms by triggering ketone bodies, mainly BHB. However, in most osteoporosis-related clinical and preclinical studies, the ketogenic diet has demonstrated the detrimental effects of inhibiting bone accumulation and damaging bone microarchitecture. In contrast, BHB is thought to ameliorate osteoporosis by promoting osteoblastogenesis and inhibiting osteoclastogenesis. The main purpose of this review is to summarize the current research progress and hope that more basic and clinical experiments will focus on the similarities and differences between ketogenic diet (KD) and BHB in osteoporosis.

## 1 Introduction

Low bone mass and damage to the microarchitecture of bone tissue, which increases bone fragility and fracture risk, are the hallmarks of osteoporosis, a systemic metabolic bone disease (1). An imbalance between bone resorption, mediated by osteoclasts, and bone generation, mediated by osteoblasts, is the fundamental process driving osteoporosis. Loss of gonadal function and aging are also major factors in the development of the disease (2). Bisphosphonates, as first-line medications for osteoporosis, are not always feasible due to their side effects (3), Instead, dietary modifications, like eating more calcium, vitamin D, and protein and following the Mediterranean diet, may have broader applicability (4–7).

The ketogenic diet is a special dietary structure with high fat, low carbohydrates, and moderate protein contents (8). The metabolic pattern shifts from glucose metabolism to fat metabolism during the ketogenic diet. Fatty acids are metabolized by the liver to produce ketone bodies, leading to nutritional ketosis. β-hydroxybutyrate (BHB), as the highest-content ketone body, not only provides energy but also participates in many metabolism processes in the body as a signal molecule, such as binding to G protein-coupled receptors (GPCRs) or histone deacetylase (HDAC) to induce anti-inflammatory and antioxidant functions. Meanwhile, the ketogenic diet has also been reported to be beneficial for various diseases, such as type 2 diabetes mellitus and neurodegenerative diseases (9–11). Recently, attention has been paid to the impact of a ketogenic diet on osteoporosis; however, BHB seems to have different outcomes for osteoporosis. In this review, we summarize the possible mechanisms underlying the actions of ketogenic diet and BHB in osteoporosis, hoping to provide directions for future research.

## 2 Overview of ketogenesis and the ketogenic diet

Woodyatt et al. first identified ketone bodies in the blood of healthy subjects on a starvation diet or a high-fat, low-carbohydrate (HF-LC) diet in 1921. Ketone bodies are intermediate products of fatty acid β-oxidation during starvation or certain pathological states. Acetyl coenzyme A, which is converted from free fatty acids, is transported to hepatocyte mitochondria through carnitine palmitoyl transferase and then converted to acetoacetate through a series of biochemical reactions. β-hydroxybutyrate dehydrogenase 1 (BDH1) reduces acetoacetate to β-hydroxybutyrate (BHB), which enters the blood circulation via the monocarboxylic acid transporter (12–14). Serum levels of BHB are quite low under normal circumstances but can reach 1–2 mM after fasting and even 6–8 mM with prolonged starvation. Meanwhile, BHB levels can spike to 20 mM in diabetic ketoacidosis (12, 15). As a major component of ketone bodies, BHB can be used by metabolically active tissues, such as the heart, brain, and muscles, where it generates cellular energy in mitochondria (13, 16). Recently, in addition to serving as an alternative fuel during starvation, more and more studies have found that BHB ameliorates oxidative stress-inflammatory states by inhibiting HDAC or binding to GPCRs (12, 16). Besides that, research on new histone post-translational modifications (HPTMs) has shown that BHB as a histone modification β-hydroxybutyrylation modified substrate regulates epigenetics (17), which may play an important role in the development of cardiovascular diseases, metabolic diseases, and other diseases (18–21).

Simultaneously, the “ketogenic diet”, which is heavy in fat and low in carbohydrates, was initially suggested by Wilder et al. at the Mayo Clinic to treat patients with refractory epilepsy by simulating a fasting condition. As such, the classic ketogenic diet was born (8, 22). With the introduction of anti-epileptic drugs, the use of the ketogenic diet declined dramatically. However, in recent decades, the ketogenic diet has drawn extensive attention again. Four variants of the ketogenic diet have emerged to provide flexibility to boost adherence: the classic ketogenic diet (cKD), the modified Atkins diet (MAD), the low glycaemic index treatment (LGIT), and the medium-chain triglyceride ketogenic diet (MCTKD) (23). The most conventional type of ketogenic diet is the classic one, which has a 4:1 ratio of fat to protein and carbs (grams to ams). Under this diet, fats provide 90% of the energy, and they are mainly derived from long-chain fatty acids, with carbohydrates and proteins providing the remaining 10%. The MCTKD provides 70%−75% of total daily energy from fat, 15%−18% from carbohydrates, and 10% from protein. Unlike the classical ketogenic diet, the MCTKD consumes more medium-chain triglycerides, which have a higher ketogenic efficiency and are more acceptable. The MAD is a much less restrictive diet with a 1:1–2:1 ratio of fat to protein and carbs (grams to ams). Here, patients can consume 1 g of carbohydrates and protein for every 1–2 g of fat consumed. During the low glycaemic index treatment, 40–60 g of carbohydrates per day is allowed, but the glycaemic index of the carbohydrate source needs to be < 50, while fat and protein intakes are not restricted. It is worth mentioning that very low-energy ketogenic therapy (VLEKT) is a novel nutritional regimen frequently employed in the management of overweight and obese individuals. VLEKT prioritizes complete calorie restrictions, permitting a total energy intake of less than 800 kcal per day, with carbohydrates limited to around 30 g/day, while fats and proteins contribute ~44% and 43%, respectively (24, 25). During the ketogenic diet, fatty acid metabolism produces ketone bodies, and ketone body levels are elevated (23, 26).

Growing evidence has demonstrated that patients with different diseases can benefit from the ketogenic diet. The ketogenic diet can assist in managing obesity and type 2 diabetes mellitus by improving fat catabolism and raising insulin sensitivity to lower blood lipids, facilitate weight loss, and improve glycosylated hemoglobin and glycaemia (10, 27). Studies have shown that the ketogenic diet can slow tumor growth, inhibit tumor metastasis, and increase sensitivity to radiotherapy or chemotherapy (28–30). It is also beneficial for the failing heart by driving increased use of ketone bodies, which can be an alternate fuel to address fuel metabolic deficits (31). Moreover, the ketogenic diet partially drives gut microbial shifts, which alleviates colitis (32, 33).

## 3 Mechanism of the ketogenic diet in bone metabolism

### 3.1 Inhibition of GH–IGF-1 axis

For normal longitudinal bone growth and bone mass build-up, growth hormone (GH) and insulin-like growth factor I (IGF-1) are necessary (34–36). IGF-1 mediates most impacts of GH on skeletal metabolism. Chondrocytes and osteoblasts in bone growth centers are targets of GH and IGF-1 action. Osteoblasts, the cells responsible for bone formation, originate from bone marrow-derived mesenchymal stem cells (BMSCs) (37). IGF-1 binds to the IGF-1 receptor on preosteoblasts and uses the phosphatidylinositol-3 kinase (PI3K)/Protein kinase B (PKB) pathway to promote stabilization of β-catenin, a signal molecule of the wingless-related integration site (Wnt) canonical pathway, which is critical for osteoblastogenesis (34, 38, 39). Stabilized β-catenin moves into the cell nucleus and attaches to members of the T-cell factor/lymphoid-enhancer factor family of nuclear proteins to regulate gene transcription, such as runt-related transcription factor 2 (RUNX2) and osterix, and promote osteoblastogenesis (38). The efficiency and rate of differentiation of preosteoblasts into mature osteoblasts control the rate of bone formation. Additionally, IGF-1 can also decrease osteoblast apoptosis and moderately promote osteoblast proliferation (40, 41).

**Figure 1:**
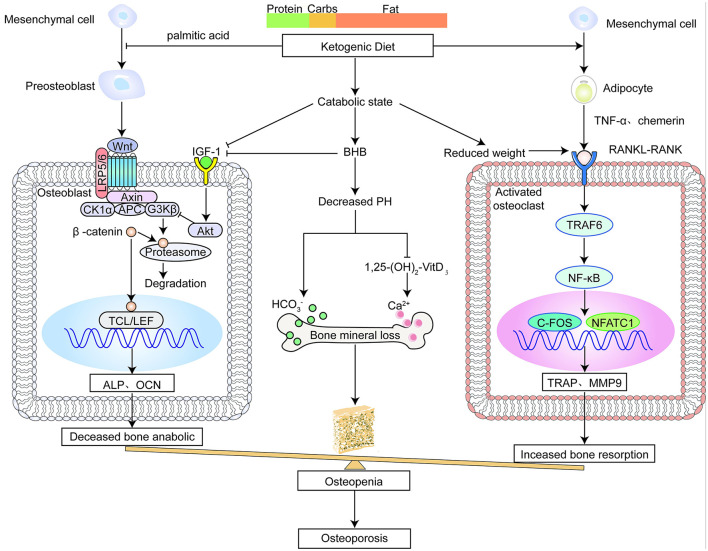
The ketogenic diet impairs bone metabolism.

GH and calorie intake primarily drive the expression of IGF-1, and IGF-binding proteins (IGFBPs) regulate its availability (36, 42). The ketogenic diet induces a catabolic state similar to starvation. This state increases the levels of IGFBPs, leading to suppression of IGF-1 activity (Figure 1) (43). Research also suggests that a HF-LC diet, as opposed to one with a higher overall calorie intake, may modulate the genetic programming of growth. Consumption of such diets leads to decreased concentrations of circulating GH and IGF-1, which ultimately impair osteogenic differentiation (44). Moreover, the increase in BHB levels caused GH axis by the ketogenic diet can create a chronic acidic environment, which may disrupt the axis (45, 46). Previous studies have found that in chronic metabolic acidosis, human serum IGF-1 concentrations are significantly reduced, and the response of IGF-1 to GH is diminished (45). The role of IGF-1 in promoting the proliferation and differentiation of chondrocytes was significantly diminished in mandibular condyles cultured under acidic conditions (46).

### 3.2 Excess of acid load

Growing evidence has demonstrated that the acid environment caused by the ketogenic diet can impair bone mineralization (Figure 1) (47, 48). Osteoblasts synthesize and secrete bone matrix, such as collagen type 1 and osteocalcin, to form dense, laminated collagen fibers. And then inorganic salts such as calcium phosphate are deposited to mineralize and harden the bone matrix. Blood pH is usually normal on a ketogenic diet, but serum bicarbonate is below normal levels, suggesting an underproduction or increased demand for associated bicarbonate ions (48). Chronic acidosis reduces bone mineral content (BMC) by speeding up the liberation of cations from bone in conjunction with bicarbonate to provide the necessary extra buffering capacity (49). This is possible due to the huge amount of potential proton buffers that bone contains. Moreover, acidosis interferes with the conversion of serum 25-hydroxyvitamin D (25(OH)D) to 1,25 dihydroxyvitamin D3 (1,25(OH)_2_D_3_), exacerbating bone damage (50).

### 3.3 Loss of weight

The ketogenic diet often causes weight loss (27), however, weight loss always results in bone loss and increased fracture risk (Figure 1) (51–53). According to the theory of mechanical homeostasis, weight loss leads to the mechanical unloading of bones, resulting in a decrease in bone mass (54). Osteocytes are particularly susceptible to biomechanical stress, and they undergo apoptosis without loading. In the absence of loading, osteoblastic activity will be suppressed and osteoclast differentiation will be encouraged (54, 55). Another possible explanation is that there are interactions among endocrine, inflammatory, and bone (56). Yu et al. found that bone resorption marker β-Crosslaps increased and bone formation marker procollagen I N-terminal propeptide (PINP) decreased after 10% body weight was lost (57). Although most studies have shown that weight loss often leads to a decrease in adipose tissue, we do not consider the inhibition of osteogenic activity caused by a decrease in estrogen and other factors caused by adipose tissue reduction.

### 3.4 High-fat diet-induced lipotoxicity

The ketogenic diet has a high fat content, which causes bone loss through local and systemic pathways (Figure 1) (58). Firstly, the current results of animal studies have shown that a high-fat diet leads to increased bone marrow tissue fat (59, 60). Bone marrow adipose tissue (BMAT) and osteoblasts are from BMSCs. The more bone marrow adipocytes there are, the fewer osteoblasts there are (61, 62). Secondly, there are numerous pieces of evidence showing that high-fat diet intake is associated with an increase in adipokines. Adipokines such as chemerin and resistin promote osteoclast differentiation (63–65), while adiponectin and leptin have a dual effect on bone cell differentiation (66–69). Generally speaking, the positive action of adipokines is not enough to counteract the negative effects, resulting in bone resorption (70, 71). What's more, a continuous high-fat diet leads to up-regulation of inflammatory genes, such as Fam3c, InhBa, Tnfsf11, and Ackr2. Inflammatory cytokines not only participate in inflammatory activities but also in bone cell differentiation (71). Previous studies indicate that inflammatory factors motivate osteoclast overactivity primarily via the receptor activator of nuclear factor-κB ligand (RANKL) signal pathway and inhibit bone marrow-derived mesenchymal stem cell differentiation into osteoblasts (72). A high-fat diet is a source of palmitic acid, which inhibits osteoblastogenesis and function and promotes apoptosis by decreasing the expression and activity of osteogenic markers such as RUNX2, alkaline phosphatase(ALP), osteocalcin, and osteomyelin (73–75). Similarly, octanoic acid derived from a medium-chain triglyceride ketogenic diet (MCTKD) leads to lower ALP and higher levels of the bone resorption marker triphosphatase (TRAP), which can adversely affect bone (76).

## 4 Mechanism of the BHB in bone metabolism

### 4.1 Regulation of osteoblastogenesis

**Figure 2:**
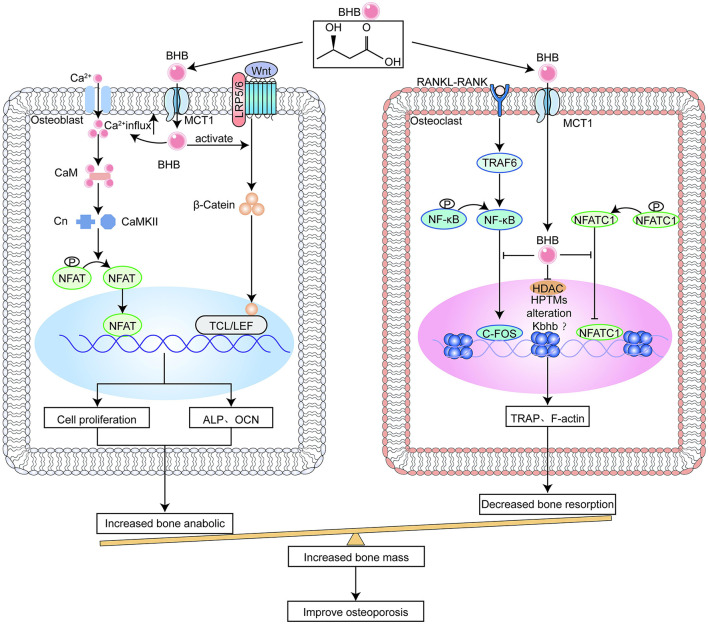
β-hydroxybutyrate (BHB) benefits bone metabolism.

BHB causes an increase in calcium inward flow, which sets off a signal cascade that promotes cell growth (Figure 2) (77). Calcium ions activate calmodulin (CaM), which regulates the nuclear factor of activated T-cells (NFAT) and calmodulin dependent kinase II (CaMKII) pathways and promotes osteoblast differentiation (78). The likely mechanism is that BHB boosts the output of adenosine 5′-triphosphatase produced by mitochondria, which causes the cell membrane to depolarize, voltage-gated calcium channels to open, and potassium channels to close (77). Calcium signaling mediates important cell cycle events, such as the re-entry of quiescent cells into G1 and the start and conclusion of the M phase (79). In addition, more energy from BHB metabolism speeds up the G1 phase of macromolecular synthesis. Considering that fatty acids make up much of the cell membrane, another logical hypothesis is that BHB supplies carbon atoms for the synthesis of fatty acids. In a manner akin to how glucose stimulates the production of insulin, a number of trophic factors are released that activate pathways that speed up the advancement of G1 (77). Apart from this, previous studies have shown that BHB promotes osteoblastogenesis *in vivo* and *in vitro*, thereby ameliorating osteoporosis (80). Thus, polyhydroxyalkanoates (PHAs) containing BHB are often used for bone repair (81). Nevertheless, it seems that the conclusion is debatable. Saito et al. concluded that acetoacetate and BHB, respectively, increase and decrease osteoblast function (82), although the exact method by which ketone bodies alter osteoblast activity remains unknown.

### 4.2 Inhibition of osteoclast differentiation

The only cells that can resorb bone are called osteoclasts, which are derived from the large, multinucleated haematopoietic cells of the monocyte/macrophage lineage. Macrophagecolony stimulating factor (M-CSF) binds to its receptor in osteoclast precursors to control cell proliferation and survival throughout differentiation. RANKL, a member of the tumor necrosis factor (TNF) superfamily, triggers many signaling cascades through its binding to receptor activator of nuclear factor-κB (RANK), thus starting the process of osteoclast precursor development and subsequent fusing into adult osteoclasts (83). The nuclear factor of activated T-cells (NFATC1), which is the primary switch that regulates the differentiation of osteoclast precursor cells, is transcriptionally activated under microgravity, leading to osteoporosis. Phosphorylated NFATC1, the inactive form of NFATC1 in the cytoplasm, dephosphorylates under the induction of RNAKL. Dephosphorylated NFATC1 enters the nucleus, attaches itself to the promoter regions of target genes, stimulates the expression of key genes for bone resorption, and activates osteoclast pre-differentiation (84). BHB or β-hydroxybutyrate methyl ester (BHBME) hinders osteoclast differentiation by reducing the active form of NFATC1 and preventing its nuclear translocation (Figure 2) (85).

On the other hand, accumulating evidence indicates that osteoclasts are activated by high levels of reactive oxygen species (ROS) and inflammatory cytokines, including TNF-α and interleukin-β (IL-1β) (86, 87). Simultaneously, previous studies have shown that BHB has a protective role in the nervous system, reproductive system, and diabetes retinopathy by inhibiting the activation of the nod-like receptor family pyrin domain containing 3 (NLRP3) inflammasome and decreasing levels of IL-1β and reactive oxygen species (ROS) production (88, 89). Recently, it has also been found that BHB inhibits inflammation and osteoclast differentiation caused by alloy particles by inhibiting apoptosis-associated speck-like protein containing a CARD (ASC) oligomerisation, assembly, and spot formation and reducing IL-1β secretion, thereby alleviating osteolysis. Notably, the inhibitory function of BHB on inflammation caused by alloy particles does not depend on the G protein-coupled receptor 109A (GPR109A) or HDAC in macrophages, but the inhibition of osteoclast differentiation and function of BHB is achieved by inhibiting HDAC (90).

## 5 Effects of the ketogenic diet on bone health

### 5.1 Ketogenic diet aggravates osteoporosis

The adverse effects of the ketogenic diet on bone health have been reported by diverse investigation groups. This section summarizes studies on the findings of the ketogenic diet on bone health and possible influencing factors. Table 1 covers research on the effects of the ketogenic diet on bone health.

**Table 1:** Summary of effects of ketogenic diet in osteoporosis.

**Type**	**Design/sample**	**Intervention**	**Duration**	**Result**	**Ref**.
Clinical research	*N =* 22; 3.5–9.8 years; Children with severe epilepsy	cKD; the ratio of fat to protein and carbohydrates is 4:1; Gradual introduction of ketogenic diet over 3 days; Provide 75% of the recommended daily calorie intake for children; Vitamin and mineral supplementation; Protein intake 1 g/kg/day.	1 year	Blood ketone bodies levels increased. Height, weight, BMI and serum IGF-1 decreased.	(43)
	*N =* 25; 7.3 ± 1.9 years^a^; Children with intractable epilepsy	cKD; the ratio of fat to protein and carbohydrates is 4:1; Vitamin and mineral supplementation; phase 1: two KD initiation protocols [traditional fasting initiation (FAST-KD); gradual initiation without fasting (GRAD-KD)]; Phase 2: EUCAL-KD (supporting weight gain at a normal velocity) and HYPOCAL-KD (restrict caloric intake).	1 year	Bone mineral content and BMI decreased.	(48)
	*N =* 20; 7–13 years; Children with intractable epilepsy	MCT; vitamin D_3_ 5,000 IU/day;	1.3–3.0 years	Bone mass decreased. Serum 1,25-(OH)2-VitD3 and calcium levels decreased.	(92)
	*N =* 40; 0.58–15.52 years; Children with epilepsy	cKD; the ratio of fat to protein and carbohydrates is 4:1; Gradual introduction of ketogenic diet over 3 days without fasting and fluid restriction; Provide 75% of the recommended daily calorie intake for children; Protein intake 1 g/kg/day; Sugar-free L-carnitine 66 mg/kg/day; Calcium 225 mg/kg/day; Vitamin D_2_ 40 IU/kg/day; Other multivitamins and mineral supplementation.	2 years	Height and weight decreased. Catch-up growth was evident in both height and weight after a year of diet discontinuation.	(93)
	*N =* 45; 0.8–17.3 years; Children with refractory epilepsy	cKD; the ratio of fat to protein and carbohydrates is 4:1; Adequate energy; Sugar-free multivitamin and calcium and potassium citrate supplementation; Protein intake 1 g/kg/day.	2 years	The average BHB serum level is 3.2 mmol/L 9% (*N =* 4) of children occurred growth retardation.	(94)
	*N =* 34; 2–17 years; Children with DRE or GLUT1-DS	cKD; Started a 1:1 ketogenic ratios subsequently increased to 2:1, 3:1, or 4:1;	More than 1 year	Serum β-OHB levels ranges from 1.8 mmol/L to 4.1 mmol/L; Fasting BHB levels moderately negatively correlated with height in the entire sample; 80% children maintained or improved their growth.	(95)
	*N =* 40; 2–16 years; Children with intractable epilepsy	MCT; cKD; cKD: the ratio of fat to protein and carbohydrates is 4:1; the ratio can be changed modified between 3:1 and 5:1; MCT: MCT energy share from 40 to 60%, carbohydrate energy share from 13 to 15%.	1 year	Height status declined. Weight status and resting energy expenditure didn't change.	(96)
	*N =* 24; 7 months−6 years and 5 months; Children with intractable epilepsy	cKD; a caloric composition of about 90% fat, 7% protein and 3% carbohydrate; Multivitamin and mineral supplementation.	1 year and 6 months	Linear growth status declined. Weight status and REE were unchanged.	(97)
	*N =* 29; 3.3–17.8 years; Patients with refractory epilepsy	cKD; the ratio of fat to protein and carbohydrates ranges from 2:1 to 4:1; Vitamin D_3_ and calcium supplementation.	More than 6 months	Bone mass density decreased. Acidic environment caused by KD altered bone and calcium homeostasis.	(98)
	*N =* 38; Mean age 6.1 years; Children with intractable epilepsy and some metabolic conditions	MAD; starting gradually with 10–30 g of carbohydrates per day; Multivitamin and mineral supplementation.	2 years	The ketone body levels were stable, near 2 mmol/L. PH was normal. Height and bone mass didn't change.	(102)
	*N =* 38; 1.5–15.5 years; Children with glycogen storage disease type 1	MCT; Complex carbohydrates, proteins and fats provide 60–70%, 15–20%, and 30% of total energy, respectively; MCT oil intakes 0.16–0.44 g/kg/day for 32–40 months	3 months	Significant improvements in skeletal muscle mass and bone mineral content.	(103)
Basic experiment	Wistar rats	CH: 9% fat, 33% protein, and 58% Carbohydrates; LC-HF-1: 66% fat, 33% protein, and 1% carbohydrates; LC-HF-2: 94.5% fat, 4.2% protein, and 1.3% carbohydrates.	4 weeks	Body length, bone mass, N-terminal propeptide of type I procollagen, expression of osteoblastogenesis transcription factors and IGF-1 decreased in LC-HF diets;	(44)
	C57BL/6J mice with bilateral ovariectomy	SD + Sham; SD + OVX; KD + Sham; and KD + OVX.	12 weeks	Cancellous and cortical bones declined in KD groups; TRAP increased and Col I decreased in KD groups.	(99)
	Sprague-Dawley rats	KD; EODKD	12 weeks	EODKD and KD both increased Blood ketone body levels and fat percentage increased; EODKD and KD both deceased bone mass and mechanical properties.	(100)
	C57BL/6J mice with bilateral ovariectomy	KD; Metformin (ip;100 mg/kg/d)		Bone mass and biomechanical properties are impaired; Metformin attenuates bone loss.	(101)

BMI, Body mass index; DRE, drug-resistant epilepsy; EODKD, every-other-day ketogenic diet; GLUT1-DS, glucose transporter type 1 deficiency syndrome; KD, ketogenic diet; LC-HF, Low-carbohydrate and high-fat; MAD, the modified Atkins diet; MCT, medium-chain triglyceride diet; OVX, ovariectomy; REE, resting energy expenditure; SD, standard diet feeding; a: age (x ± SD).

Several studies are related to bone abnormalities after a ketogenic diet in children and adolescents. The alterations associated with a ketogenic diet contributing to the failure of bone mineral build-up include abnormalities in altered vitamin D levels, acid loading, decreased weight, increased levels of blood BHB, and indirect or direct disruption of the GH axis (43, 91–98). In these studies, it has been mentioned that a ketogenic diet inevitably leads to growth retardation in children, but there are also studies indicating that after stopping the ketogenic diet for a period of time, children will experience significant catch-up growth (93, 94). There is poor clinical research on the potential impacts of the ketogenic diet on adult bone health, and there is not much data on the long-term risk of osteoporosis and a ketogenic diet. However, due to the close correlation between the development of osteoporosis and the accumulation of peak bone mass in adolescence, there are reasons to believe that a ketogenic diet is harmful to the development of osteoporosis.

In addition, there are several animal model studies that support evidence of bone impairment induced by a ketogenic diet (Table 1). The ketogenic diet disrupted cortical bone mass and bone microstructure in a similar way to ovariectomy (OVX) in mice, mediated by promoting osteoclast differentiation in the research of Wu et al., while Xu et al. thought that a ketogenic diet suppressed osteoblast activity by reducing weight and increasing body fat percentage, leading to reduced bone formation (99, 100). But it has been reported that metformin alleviates the cancellous bone loss caused by the ketogenic diet (101). Rats on a high-fat and low-carbohydrate diet demonstrated visceral fat accumulation, and adipocytes in bone marrow were significantly increased. The expansion of bone marrow adipose tissue (BMAT) inhibits osteoblastogenesis, exacerbating bone loss (44).

### 5.2 Ketogenic diet does not worsen osteoporosis

Nevertheless, some studies suggest that a ketogenic diet has no unfavorable impacts on bone health. No adverse effects on bone mass or longitudinal growth were noted in a study using MAD. Stable pH, proper serum ketones levels, and more calorie and protein consumption than a classical ketogenic diet may explain the lack of detrimental effects on bone development (102). In a separate trial utilizing MCTKD to treat children with glycogen storage disorder, it revealed enhancements in the patients' BMC and skeletal muscle mass. This may be due to MCTKD's ability to improve hyperlactatemia, which can facilitate osteoclastogenesis and impede osteoblastogenesis. Another reason may be that MCTKD can enhance lipolysis and ameliorate fat inflammation (103). A thorough evaluation found no significant bone density changes in adults receiving a ketogenic diet. Only female participants who dropped 10% of their weight had bone mechanical unloading and increased bone turnover due to energy restrictions and quick weight loss, but osteoporosis risk did not increase (91).

## 6 Effects of BHB on bone health

### 6.1 BHB improves osteoporosis

It is generally believed that BHB reduces bone loss and improves osteoporosis by promoting osteoblastogenesis and inhibiting osteoclast formation. Table 2 covers research on BHB's effects on bone health.

**Table 2:** Summary of applications of BHB in osteoporosis.

**Type**	**Intervention model**	**Intervention material**	**Result**	**Ref**.
*In vivo*	Wistar rats with bilateral ovariectomy	30, 150, 750 mg/kg/d; BHB (po;12 weeks)	Osteoblast differentiation increased. Bone mineral density and bone mechanics increased.	(80)
	ICR mice with hind limb unloading	50,100,200 mg/kg/d; BHB (po;4 weeks)	Serum calcium levels decreased. Bone mineral density increased.	(85)
	C57BL/6J mice with osteolysis surgery	20% (v/v) 1, 3-butanediol in drinking water (2 weeks)	Osteoclast differentiation and function decreased. Bone mineral density increased. Osteolysis decreased.	(90)
*In vitro*	MC3T3-E1 cells	0.005, 0.01, 0.02, 0.05, 0.1 g/L BHB	Osteoblast differentiation increased.	(80)
	MC3T3-E1 cells	0.05, 0.5, 5 mmol/L Acetoacetate; 0.05, 0.5, 5 mmol/L BHB	Osteoblast functions, respectively, increased and decreased under acetoacetate and BHB.	(82)
	RAW 264.7 cell line	1, 10 mmol/L BHB	Activated NFATC1 decreased. Osteoclast differentiation decreased.	(85)
	Osteoclasts	2,4,6 mol/L (R)–BHB or (S)–BHB	HDAC decreased. Osteoclast differentiation decreased.	(90)

HDAC, histone deacetylase; NFATC1, the nuclear factor of activated T-cells 1.

The research conducted both *in vivo* and *in vitro* supports the potential benefits of BHB in enhancing osteoblastogenesis and anti-osteoporosis in rats undergoing bilateral OVX (80). However, the specific mechanism by which BHB promotes osteoblastogenesis needs further exploration. Furthermore, some studies show that poly(3-hydroxybutyrate) composed of BHB induces ectopic bone formation after implantation into the back muscles of cats, minipigs, and rats (81, 104). Another possible reason is that BHB activates signal transduction pathways for osteoblast proliferation by triggering rapid calcium ion flow into cells (77).

Besides that, Cao et al. found that BHB suppresses osteoclast differentiation and improves osteoporosis under microgravity conditions by inhibiting NFATC1 transcription (85). Wu et al. administered 1,3-butanediol (a BHB precursor) to mice undergoing bone osteolytic surgery. It was found that the bone density of mice increased and the activity of osteoclasts was inhibited. The specific mechanism is that BHB ameliorates osteolysis by inactivating inflammasomes and inhibiting the development and function of osteoclasts (90).

### 6.2 BHB aggravates osteoporosis

However, as mentioned earlier, acetoacetate and BHB, respectively, up-regulate and down-regulate mineralization and ALP activity in osteoblasts (82). It may partly explain the bone degeneration in diabetics. The ratio of BHB to acetoacetate rises as diabetes worsens (105), and it is possible that BHB has a stronger inhibitory effect than the promotional effect of acetoacetate on bone formation. This suggests that ketone bodies may also be the cause of diabetic osteoporosis (82).

## 7 Conclusion

The effects of the ketogenic diet on osteoporosis remain controversial. Most studies suggest that ketogenic diets may impair bone health and increase the risk of osteoporosis by suppressing the GH-IGF-1 axis, acid load, weight loss, and lipotoxicity (Figure 1). However, other clinical studies have shown that some types of ketogenic diets do not damage bone mass. This may be because MAD does not severely restrict protein and calorie intake. In addition, medium-chain triglycerides is thought to improve bone mineral accumulation by inhibiting osteoclastogenesis and promoting osteoblastogenesis. However, the sample size of these studies was small and the follow-up period was limited. BHB improves osteoporosis by inhibiting osteoclastogenesis and promoting osteoblastogenesis through inhibition of the RANKL signaling pathway and promotion of calcium in-flow, respectively (Figure 2). However, it has also been shown that BHB may inhibit ALP activity and thus inhibit mineralization. This may be one of the causative factors of diabetic osteoporosis.

Most of the clinical studies of the effects of ketogenic diets on bone health have targeted populations such as children with refractory epilepsy, glycogen storage disorders, and other disorders and obese populations (Table 1). The majority of clinical studies have shown adverse effects of ketogenic diets on children's bones. Childhood and adolescence are critical stages for bone mass accumulation. Impaired bone mass accumulation or inadequate nutritional intake during this period may cause a decrease in peak bone mass in adulthood, which may increase the risk of osteoporosis and fractures (106). Therefore, during the application of the ketogenic diet in children, regular follow-ups should be performed to monitor growth, including annual growth rate, height, weight, bone density, growth hormone, vitamin D, and blood calcium levels. Adequate vitamin D, calcium, and protein should also be taken during treatment (107, 108). Adequate sunlight hours and physical activity are also important for bone mass accumulation. When using the ketogenic diet for weight loss in obese and overweight people, it should be ensured that the rate of weight loss is within the normal range so as not to cause an increase in bone turnover due to rapid weight loss.

The effects of ketogenic diets and BHB on osteoporosis are the result of a multifactorial overlap. Conflicting results and the lack of precise molecular and biochemical mechanisms of action provide ample opportunities for future research. Emerging avenues of research, including BHB-induced β-hydroxybutyrylation, may play a role in modulating osteoporosis. Current clinical studies have focused on adolescent subjects, and there is a lack of research in the elderly population, where the prevalence of osteoporosis is significantly higher. More targeted, longer-term, and broader clinical studies, including precursor substances that induce ketone bodies, or modified ketogenic diet formulation interventions for different populations, may help to analyze the relationship between ketone bodies and osteoporosis.
